# Nursemaid’s Elbow With Transient Wrist Drop in a Toddler: An Atypical Presentation

**DOI:** 10.7759/cureus.115268

**Published:** 2026-08-26

**Authors:** Christeen Mina, Charbel Tannous, Nancy Matar

**Affiliations:** 1 Emergency Department, Lebanese American University Medical Center, Beirut, LBN; 2 Emergency Medicine Department, Lebanese American University, Beirut, LBN

**Keywords:** emergency medicine and trauma, nursemaid's elbow, posterior inter-osseous nerve, pseudoparalysis, radial head subluxation, radial nerve, trauma pediatric, wrist drop

## Abstract

Radial head subluxation, commonly known as nursemaid's elbow, is a frequent upper extremity injury in young children and typically presents with refusal to use the affected arm without focal distal motor dysfunction. We report an atypical presentation in a previously healthy two-year-and-10-month-old girl who was brought to the emergency department after an unwitnessed event with no clearly identified mechanism of trauma. She held her right elbow flexed against her chest in a posture suggestive of radial head subluxation, and additionally refused to let go of her wrist and demonstrated a wrist-drop posture, absence of active finger movement, mild wrist edema, and tenderness predominantly over the wrist. An initial reduction using a hyperpronation maneuver produced no palpable or audible click and no clinical improvement. Because of the atypical distal findings, further manipulation was deferred, and radiographs of the affected extremity were obtained, showing no acute fracture or dislocation. A second hyperpronation reduction was subsequently performed, during which a distinct palpable and audible click was appreciated. Immediately afterward, active wrist extension returned and the wrist-drop posture resolved. The patient reached for and successfully held an object, and within approximately two minutes demonstrated full spontaneous movement of the affected upper extremity without residual tenderness, edema, or apparent motor deficit. She was observed for 20-30 minutes and subsequently discharged home. This case highlights an unusual neurologic-appearing presentation of radial head subluxation. The rapid resolution following successful reduction suggests a close association between the subluxation and transient distal dysfunction; however, pain-mediated pseudoparalysis and transient radial motor nerve dysfunction cannot be reliably distinguished in a young child.

## Introduction

Radial head subluxation, commonly known as nursemaid's elbow or pulled elbow, is a frequent upper extremity injury in young children, particularly between one and four years of age. It classically occurs following longitudinal traction on the arm and typically presents with sudden refusal to use the affected extremity, with the child holding the arm protectively. Diagnosis is predominantly clinical, and successful closed reduction usually results in rapid restoration of normal upper extremity use [1].

The epidemiology of radial head subluxation reflects the developmental anatomy of early childhood. In a retrospective series of 2,331 cases, Irie et al. found that the injury occurred predominantly in young children, with the highest incidence around two years of age. Although forcible traction is considered the classic mechanism, it accounted for only approximately half of the cases in that series [2]. Furthermore, Sacchetti et al. demonstrated that the absence of a classic history of upper extremity traction should not exclude radial head subluxation when the clinical presentation is otherwise compatible [3].

Atypical presentations can create diagnostic uncertainty, particularly when the mechanism is unwitnessed or when examination findings suggest a more distal musculoskeletal or neurologic abnormality. A wrist-drop-like posture may raise concern for impaired wrist extensor function, while posterior interosseous nerve (PIN) dysfunction more characteristically produces weakness of finger and thumb extension. However, in preverbal or very young children with limited cooperation, distinguishing true motor dysfunction from pain-mediated pseudoparalysis may be difficult, and such findings should not be assumed to represent a confirmed neurologic lesion.

We report the case of a two-year-and-10-month-old girl with an unwitnessed injury who presented with findings suggestive of radial head subluxation accompanied by a wrist-drop-like posture and absence of active finger movement. The distal motor abnormality persisted after an unsuccessful initial reduction and despite radiographs showing no acute fracture or dislocation, but resolved immediately following a subsequent successful reduction. The unusual feature of this case was the transient distal motor dysfunction that resolved with successful reduction, rather than simply an atypical presentation of radial head subluxation. This case highlights the importance of careful distal examination, appropriate evaluation of neurologic-appearing findings, and reassessment following reduction, while recognizing that the observed deficit cannot definitively distinguish transient nerve dysfunction from pain-mediated pseudoparalysis.

## Case presentation

A previously healthy two-year-and-10-month-old girl with no significant medical or surgical history was brought to the emergency department by her parents after being found unwilling to move her right upper extremity. The event preceding her symptoms was unwitnessed, and no definite mechanism of trauma or history of longitudinal traction could be identified.

On presentation, the child held onto her right wrist, with the right elbow flexed against her chest, and refused to let go of the wrist. The affected wrist additionally demonstrated a wrist-drop-like posture, and she did not demonstrate active finger movement during the initial examination. Mild edema was present over the wrist, without ecchymosis or obvious deformity. Palpation and movement elicited tenderness predominantly over the wrist, with comparatively mild tenderness around the elbow. The radial pulse was palpable, and capillary refill was normal. Formal sensory assessment was limited by the patient's age and ability to cooperate.

Given her age and characteristic positioning of the elbow, radial head subluxation was initially suspected despite the absence of a witnessed traction mechanism. A reduction was attempted using a hyperpronation maneuver. No palpable or audible click was appreciated, and there was no clinical improvement. The patient continued to maintain the wrist in a dropped position and did not demonstrate active finger movement.

Because of the atypical presentation, including the wrist-drop-like posture, absence of active finger movement, wrist edema and tenderness, unknown mechanism of injury, and unsuccessful initial reduction, no further forceful manipulation was attempted at that stage. No analgesia or sedation was administered. Plain radiographs of the right elbow, forearm, wrist, and hand were obtained to evaluate for an associated traumatic injury.

**Figure 1:**
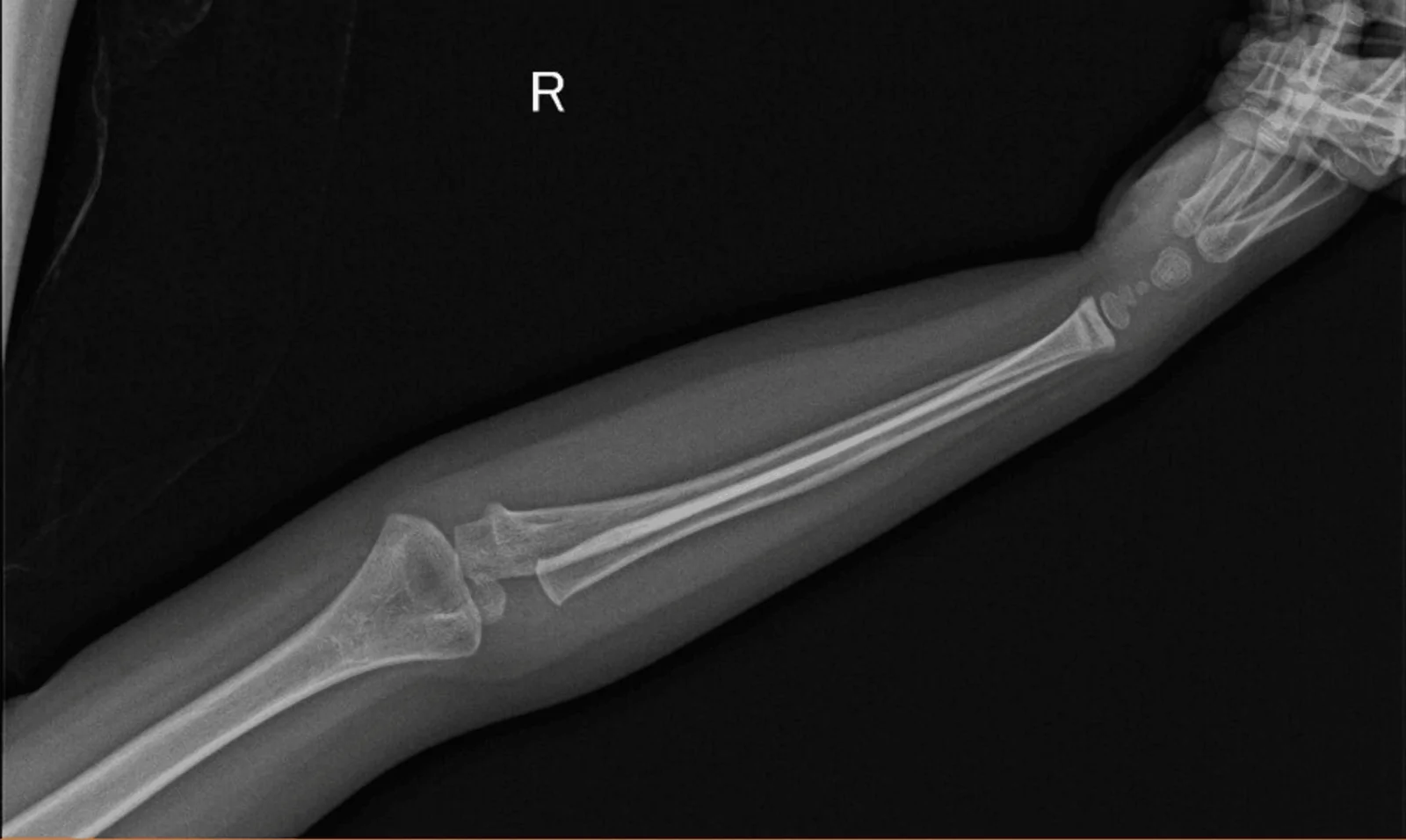
Lateral Radiograph of the Right Elbow No acute fracture or dislocation is identified, with preserved alignment.

**Figure 2:**
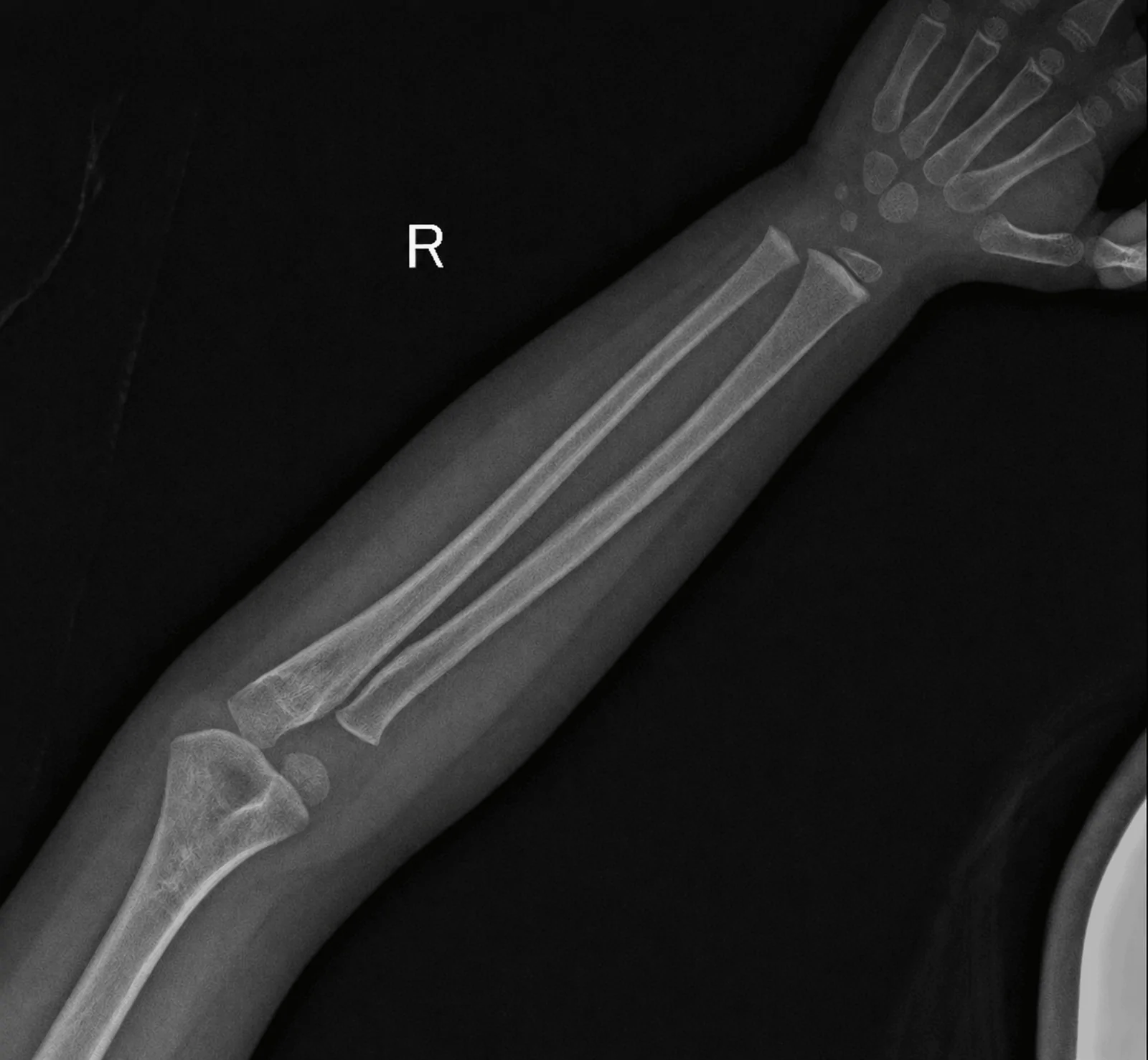
Anteroposterior Radiograph of the Right Forearm The right forearm, including the elbow and wrist, demonstrates no acute fracture or dislocation, with preserved osseous alignment.

**Figure 3:**
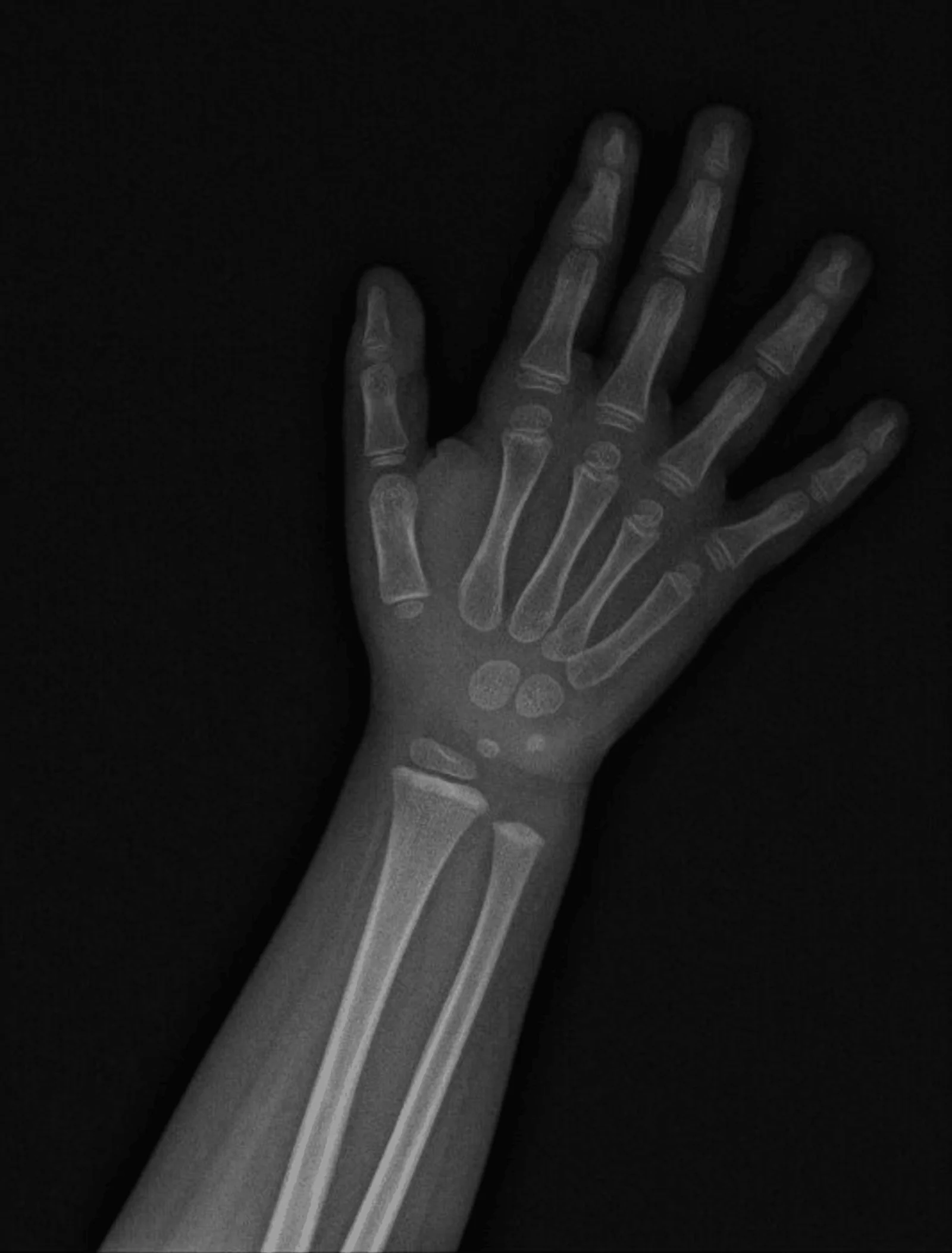
Anteroposterior Radiograph of the Right Hand and Wrist No acute fracture is identified, and osseous alignment is preserved.

Radiographs demonstrated skeletal immaturity without evidence of an acute post-traumatic fracture. Joint spaces were preserved, osseous structures were adequately aligned, and no dislocation was identified. Representative radiographs are shown in Figures 1-3.

The orthopedic team was consulted after imaging and, given the atypical presentation and persistent concern for an occult injury despite negative radiographs, recommended cast immobilization for a possible physeal (growth plate) injury. Before the cast was applied, radial head subluxation remained a clinical consideration, and a second reduction was attempted using the same hyperpronation maneuver. During this attempt, a distinct palpable and audible click was appreciated at the elbow.

Immediately following the second reduction, the wrist-drop-like posture resolved, with restoration of active wrist extension. The patient reached for an object and was able to grasp and hold it successfully without dropping it, demonstrating restoration of functional finger movement. Within approximately two minutes, she demonstrated full spontaneous movement of the affected upper extremity without recurrence of the dropped wrist posture or apparent distal motor impairment. Repeat examination revealed no residual tenderness or appreciable edema.

The patient was observed in the emergency department for approximately 20-30 minutes, during which normal use of the affected extremity was maintained without recurrence of symptoms. She was subsequently discharged home in stable condition. No subsequent follow-up was ordered.

## Discussion

Radial head subluxation is a common pediatric upper extremity injury and is particularly frequent in early childhood. The usual presentation consists of sudden refusal to use the affected arm after longitudinal traction, with the extremity held protectively and without a focal distal motor deficit [1]. Large series have confirmed that the condition is concentrated in young children, with peak incidence around two years of age [2]. Importantly, the absence of a witnessed traction event does not exclude the diagnosis. Sacchetti et al. showed that children with radial head subluxation may present without the classic history when the clinical examination is otherwise compatible [3]. This was relevant in our patient, whose symptoms followed an unwitnessed event with no clearly identifiable mechanism.

Several features of the present case differed from the usual presentation. Although the child's elbow posture initially suggested radial head subluxation, the wrist-drop-like posture, absence of active finger movement, mild wrist edema, and greater tenderness at the wrist than at the elbow raised concern for an alternative or additional traumatic injury. The first reduction attempt also failed to produce a palpable or audible click or any clinical improvement. In this setting, repeated manipulation without reassessment could have risked overlooking an occult fracture, dislocation, or neurologic injury. Imaging was therefore obtained before a second reduction attempt.

The most unusual finding was the transient distal motor dysfunction. A wrist-drop-like posture may raise concern for dysfunction of the radial motor pathway, but precise neurologic localization from the examination of a distressed toddler is difficult. The PIN, the deep motor continuation of the radial nerve, supplies most of the finger and thumb extensors. Isolated PIN neuropathy therefore typically causes marked weakness of finger and thumb extension while at least some wrist extension is usually preserved because important wrist extensors receive innervation proximal to the PIN [4]. The combination of a dropped wrist posture and absence of active finger movement in our patient therefore cannot confidently be localized to an isolated PIN lesion. Formal sensory assessment was also limited by age and cooperation, and electrodiagnostic testing was not clinically indicated after the observed deficit resolved immediately following reduction.

An anatomic relationship between radial-head pathology and PIN dysfunction has been described in children. Tubbs et al. reported PIN compression in a child with radial malunion and recurrent radial-head instability [5]. Ruchelsman et al. similarly described persistent PIN palsy associated with a chronic radial-head dislocation in a pediatric Monteggia fracture-dislocation [6]. These reports involve substantially more severe and persistent structural abnormalities than the transient radial head subluxation of nursemaid's elbow and therefore do not establish the same mechanism in the present case. They do, however, demonstrate that the PIN may be affected by displacement or instability involving the radial head. A transient traction- or compression-related radial motor phenomenon is therefore anatomically conceivable, but it cannot be demonstrated from the findings of this case.

An important alternative explanation is pain-mediated pseudoparalysis. Young children may respond to musculoskeletal pain by avoiding movement of an affected limb, creating the appearance of profound motor weakness. Orta et al. reported a two-year-old child with an unwitnessed event who presented with striking bilateral upper extremity pseudoparalysis; bilateral nursemaid's elbows were ultimately diagnosed, with rapid restoration of upper extremity function after reduction [7]. Although that presentation differed from the focal wrist-drop-like appearance in our patient, it demonstrates that radial head subluxation can produce neurologic-appearing loss of function in a young child. Pain, distress, and limited cooperation may therefore have contributed to the apparent motor deficit in our patient. This possibility is particularly relevant because no analgesia or sedation was administered, making it difficult to distinguish pain-mediated inhibition from true transient motor dysfunction on the initial examination.

The temporal relationship between successful reduction and recovery is particularly notable. The first hyperpronation maneuver produced neither a palpable nor audible click and resulted in no improvement. After radiographs excluded an acute fracture or dislocation, the second hyperpronation maneuver produced a distinct palpable and audible click, followed immediately by restoration of active wrist extension. The child then reached for and securely held an object, demonstrating functional finger movement, and within approximately two minutes demonstrated full spontaneous use of the affected extremity. Hyperpronation and supination-flexion are established reduction techniques for radial head subluxation. A systematic review and meta-analysis by Bexkens et al. found hyperpronation to have a lower first-attempt failure rate than supination-flexion, although the included studies had methodological limitations [8]. An unsuccessful initial maneuver therefore does not by itself exclude radial head subluxation when the subsequent clinical course supports successful reduction.

The immediate recovery supports a close temporal association between successful reduction and resolution of the distal motor abnormality, but it does not establish the underlying mechanism. A transient radial motor phenomenon is possible given the wrist-drop-like appearance and rapid recovery after reduction. Conversely, pain-mediated pseudoparalysis could produce apparent weakness without structural nerve dysfunction and may be difficult to distinguish from true motor impairment in a child younger than three years. The limited formal neurological examination and absence of electrodiagnostic testing prevent definitive differentiation between these explanations. Describing the finding as wrist-drop-like distal motor dysfunction temporally associated with radial head subluxation and successful reduction is therefore more appropriate than diagnosing a radial nerve or PIN palsy.

This case also emphasizes the value of serial examination. The atypical distal findings and lack of response to the first reduction appropriately prompted imaging before further manipulation. Reassessment after the second reduction was equally informative: immediate restoration of wrist extension, successful grasp of an object, and full spontaneous use of the extremity provided clinically meaningful evidence that the functional deficit resolved in close temporal association with successful reduction.

This report has several limitations. The patient's young age, distress, and limited cooperation restricted formal motor and sensory examination and prevented precise neurologic localization. Pain-related inhibition may also have affected the initial assessment, particularly because no analgesia or sedation was administered. No electrodiagnostic or advanced imaging studies were performed because the symptoms resolved completely following reduction and there was no clinical indication for further investigation. In addition, no outpatient follow-up was obtained. Consequently, although complete functional recovery was observed during the emergency department observation period, sustained neurological recovery and absence of recurrence cannot be confirmed.

Despite these limitations, the observed sequence of persistent wrist-drop-like distal motor dysfunction before successful reduction, a palpable and audible reduction click, immediate restoration of active wrist extension and functional grasp, and full spontaneous use of the extremity within approximately two minutes represents an unusual presentation of an otherwise common pediatric injury. Clinicians encountering atypical distal motor findings in a young child with suspected radial head subluxation should carefully evaluate for alternative injury, avoid assuming a specific neurological mechanism, and reassess distal motor function after reduction. The rapid resolution of neurologic-appearing findings may be clinically informative, but should be interpreted as a temporal association rather than evidence of a confirmed nerve lesion.

## Conclusions

Transient distal motor dysfunction is not a typical feature of nursemaid's elbow. In this case, a wrist-drop-like posture with absence of active finger movement resolved immediately after a clinically successful reduction, demonstrating a close temporal association between successful reduction and restoration of distal motor function. The available examination cannot determine whether the presentation represented pain-mediated pseudoparalysis, transient radial motor dysfunction, or a combination of both, and no specific neurologic mechanism can be confirmed.

Clinicians evaluating suspected radial head subluxation should document distal motor and neurovascular function before and after reduction and should reconsider the diagnosis when the mechanism is unclear, the examination is atypical, or an initial reduction is unsuccessful. Appropriate imaging before repeated manipulation and careful post-reduction reassessment can help exclude associated injury and confirm restoration of function. Atypical neurologic-appearing findings should therefore be interpreted cautiously and in the context of the overall clinical course.
